# Webinar among Indonesian academics during Covid-19, embracing the audiences

**DOI:** 10.1371/journal.pone.0265257

**Published:** 2022-03-16

**Authors:** Setiawan Priatmoko, Billal Hossain, Wuri Rahmawati, Susilo Budi Winarno, Lóránt Dénes Dávid

**Affiliations:** 1 Doctoral School of Economic and Regional Sciences, Hungarian University of Agriculture and Life Sciences (MATE), Gödöllő, Hungary; 2 Department of Management, STIE Pariwisata API, STIEPARAPI, Yogyakarta, Indonesia; 3 Faculty of Social Politic, Department of Communication, Aisyiah University, UNISA, Yogyakarta, Indonesia; Satyawati College (Eve.), University of Delhi, INDIA

## Abstract

This research aims to find out the phenomenon of webinar competition from the viewpoint of the audience. Covid-19 pandemic makes webinars a means for knowledge dissemination. Many events offered turned out to be a tight competition among organizers and caused a different response for the audience. Academics participants’ responses had never been known in depth so that they could be the basis for determining the strategy for the organizers. Using quantitative data through online surveys to further interpreted with the help of previous literature. The independent variables gender, age, frequency, cost, and place are used to determine their effect on loyalty, which is represented by the length of duration in participating in each webinar. The effectiveness of webinars as a means of disseminating ideas in the pandemic era still faces various challenges. Among academics, the loyalty at the webinar event is influenced by gender and age. It is important for organizers to effectively communicate to webinar participants so that they get the message they want to convey.

## Introduction

A webinar or website seminar is a form of seminar using a website platform. A webinar is a seminar that is conducted over the Internet [1]. In general, holding a webinar involves several parties in different dimensions of space and time at the same time. In the Covid-19 pandemic situation when social and physical restrictions were an obstacle then the webinar was considered as one solution to continue to establish communication and dissemination of the purpose of a scientific seminar or meeting.

The situation in Indonesia during the Covid-19 pandemic is also not much different from other countries. Prevention of virus transmission with social restrictions is carried out massively, including in the fields of education and science. Restrictions on physical gatherings in classrooms and on-campus spaces are nationwide in effect, teaching and scientific forums are turning online. The challenges and obstacles faced by scholars in Indonesia in this pandemic situation need to be known to get a deeper understanding approach about the education issue. Webinar as one of the popular instruments used in the dissemination of knowledge during a pandemic is a phenomenon that has now become a daily social activity among scholars.

The rise of the webinar was driven by the adoption of the emergency response period with the issuance of Indonesian Government Regulations PP No. 21/2020. Some regions in Indonesia even started to restrict human movements before the regulation was enacted. One of these limitations impact is the absence of physical activity on the campuses. Luckily, this helped encourage academics to join webinar events that are often open to the public and academics held by other parties. Specifically for academics and higher degree students in Indonesia, webinars have become the dominant activity within the limitations of physical encounters in the pandemic era. Nowadays, webinars in Indonesia have become a medium for launching and introducing new products to consumers. What webinar organizers do is use online knowledge-sharing events by incorporating their product message into the webinar event.

In Indonesia, webinars are now becoming popular for disseminating various information. Various government institutions and private sectors use the webinar platform to convey the messages they want to spread. To put it another way, online meeting platforms, which are commonly utilized in education, are also employed by other stakeholders such as government and commercial institutions. They spread their event information via WhatsApp messenger.

The objective of the research is to find out the random webinar events offered among academics through a poll. It is believed that the results of the polls will reveal the state of the webinar’s activities for the participants. Understanding the webinar with a conceived as a product will help us more easily analyse its phenomenon. At the webinar’s participant level, we also examined the effects of gender, age, frequency of attending the webinar, cost of internet, and place of accessing the webinar toward the duration intentions of attending the webinar. Thus, the opinions that are known deeper and subsequently processed can be used as the basis for further implementation of various online meeting information dissemination activities and their challenges.

## Literature review

The application of social distancing rules that limit the gathering of humans physically encourages changes in the form of human encounters. The impetus for implementing social distancing is influenced by attention to personal hygiene practices[2]. Health issues and the risk of danger of transmission of novel coronavirus disease pandemic disease are the main causes of limiting physical encounters. Coronavirus can be transmitted through droplets and close contact and is known as a highly infectious [3]. Based on the ease of transmission of the disease, the community conducted a meeting through the internet. In academics, meetings in classrooms or indoor meetings instantly turn into meetings via the internet.

Teaching and dissemination of this knowledge are carried out through webinar schemes during the pandemic. Opportunities in the webinar can be created among educators and learners in online mode to experience different levels of interaction [4]. Despite webinars highly useful tools, the instructional design during the webinar aspects, the content of the training or how the webinar can be used tend to be deemphasize [5]. The challenges in conducting webinars are still not considered to be equal compared to the ease of physically meeting in class or real meeting rooms [6].

Principles in webinar design should aware of considerations applicable to adult learning which are clear objectives and content relevant to practice to make understanding of knowledge easier [7]. It is much more difficult to know the group’s interests and experience through webinars, so its need the right level in terms of relevance and interest. The restriction for the presenter is also more challenging that the presentation has been made beforehand and therefore cannot be changed easily to suit the audiences’ needs [8]. Thus, getting attention from viewers who will be the target of disseminating information becomes a challenging situation.

The number of campuses, academics, study groups, and business entities that disseminate knowledge, findings, training, and even promotion to the public through webinars in the pandemic era is a new phenomenon. The general promotion is using digital posters distributed via the Whatsapp messenger application. According to the Hootsuite [9], WhatsApp Messenger is the biggest instant messaging service in Indonesia. The uses for its messenger service platform because online chatting has proved to be a strong promotion and marketing hub in this century [10]. The webinar offers are distributed and forwarded randomly via WhatsApp, and this is a common phenomenon in Indonesia today.

The webinar organizer hopes that a big number of people will attend. This is a tough challenge considering that in this pandemic era almost all institutions (government and private sector) also hold webinar events with the same target audience, which is the public. Although webinar events are more often shared and knowledge dissemination, the decision to attend the event can be likened to a desire to make a purchase. Consumer purchasing decision refers to the final decision a consumer takes after considering all the factors [10]. According to the data collected by the CMO Council, 71% of internet users are more likely to purchase from a brand that they are following on a social networking [11]. Content in webinar events is the main issue in the promotion conducted by the organizer. Thus, the content marketing concept is based on the assumption that when enterprises provide valuable information for the customer, they can expect a useful customer response [12], including webinar’s content.

In the competition to get participants/audience webinars, it would be important to market the activities of the webinar become viral. Viral marketing is a model of word of mouth marketing using the Internet media [13]. More positive reviews on virtual communities creates greater trust in a brand, as people consider social eWOM/ electronic Word of Mouth to be their reference to avoid risks from consuming a product they know nothing about [14].

The next challenge in disseminating knowledge in webinars is to ensure that the content delivered can be absorbed optimally by participants because it is even more satisfying in face-to-face instruction compared with the webinar [5]. There are several factors that affect the audience’s response to pay attention among them are usefulness, service quality, information quality, and system quality [15]. Content in a product in this case is a webinar event that also acts as a tactic to build and sustain relationships to target audience [16]. The audience’s commitment to pay attention to the content delivered at the webinar also illustrates their loyalty to the event product. Loyalty intention is a customer’s behavioural commitment to repatronize a certain service provider or a firm [17]. Because the information presented did not reveal an "optimal" webinar length, educational technologists and practitioners can continue to rely on trial and error [18]. Furthermore, the meaning of loyalty will be limited to grouping the duration of the audience’s ability to stay and pay attention to a webinar.

Gender differences exist in the preferences of using online platforms [19]. In another study in the USA, it was found that in the delivery of course content, age and gender had no significant effect [20]. A study by Alfadda and Mahdi show a negative correlation between the students’ gender and their acceptance of using Zoom for learning [21]. Another study stated that the terminology of internet costs is in the context of uploading and downloading data speeds [22]. On the other hand, technical internet connectivity issues related to the speed of the internet packages cost also affect learning via learning modalities [23]. However, response to the randomly webinar event toward gender, age, intensity, and internet cost has not been studied for a specific group of respondents in developing countries.

There is significant evidence across these researchs that distant information dissemination via randomly advertised webinars is influenced by a variety of factors. However, there is a lack of research on the information of audiences’ impressions about attending its random webinars, related resources, and its event. The factors that influence the duration of attending the webinar related to certain demographic data (gender, age, frequency, cost, and place) among higher degree students in developing countries have not been investigated. Based on the literature presented above, the following hypotheses are also proposed:

**Hypothesis 1 (H1):** Gender has a positive effect on the duration of attending the webinar.
**Hypothesis 2 (H2):** Age has a positive effect on the duration of attending the webinar.
**Hypothesis 3 (H3):** Frequency of attending webinars has a positive effect on the duration of someone attending the webinar.
**Hypothesis 4 (H4):** Cost considerations have a positive effect on the duration of someone attending the webinar.
**Hypothesis 5 (H5):** Place to access the internet did not have a significant effect on the duration of someone attending the webinar.

Finally, this research will not only report descriptively the answers of academics in developing countries but will also investigate the factors that determine how long they stay on the webinar screen.

## Materials and methods

This research presents a quantitative approach using the survey method for its primary data collection through an online survey. The steps are divided into several stages. First, we did an elaboration of previous research related to webinar activities through a literature review. secondly, we assume that the webinar activity offered by various parties is a product that has the objective of gaining consumers which is the audience. Thus, there are two main aspects in these webinar event activities, namely from the audience response to the webinar event and from the duration aspect in participating in the event. This will further determine the questions given in the survey form.

### Data collection

In taking the sample of this study using a stratified systematic random sampling technique. On this survey, we only conducted a sample of those who study in the field of social science in Indonesia. The selection of participants in this group is because the number of study programs in the field of science-engineering is less than the field of social science-humanities, respectively 11,734 (41 percent) and 16,817 (59 percent) [24]. The other reason is that one of the most interesting aspects of social science study student is their research on and with actual people in the real world. For the social studies is always changing, they will be eager to come up with new social questions to ask or current social concerns and events to investigate [25]. It suggests they are more receptive to a fresh idea that has been disseminated through a random promoted webinar.

Data is collected through WhatsApp (WA) messenger group. WA is the most popular platform for communication between faculty and their students outside classrooms [23]. The use and ownership of WA messenger is very popular in Indonesia instead of a mailing list. The responses given by respondents are also relatively fast and trusted by prospective respondents because the sender of the questionnaire is a person who has been validated or known by other members of the WA messenger groups. Moreover, Based on Statista data, Indonesia is the country with the third most WhatsApp users in the world after India and Brazil, the number reaching 84.8 million users in June 2021[26]. The second reason for using WA groups in the survey is that Indonesians like to form groups based on shared interests or backgrounds, such as hobbies, work, or hobbies, as well as academics.

The questionnaire was created in digital format and distributed using the Google Forms. The data in this survey were obtained from WhatsApp Messenger/ WA groups that contain faculty members, researchers, and higher degree students in Indonesia. From several WA groups with a total of 448 members, 211 responses were obtained. With a confidence level of 95% and a margin of error (MOE) of 5%, the minimum number of samples was 207. In this study, the questionnaire returned 211 responses. We use the formula:

$$\frac{\frac{z^{2}\times p(1-p)}{e^{2}}}{1+(\frac{z^{2}\times p(1-p)}{e^{2}N})}$$

Description

N = Population Size

e = Margin of Error (as a decimal)

z = Confidence Level (as a z-score)

p = Percentage Value (as a decimal)

The data acquisition can be seen in the following table:

The number of 211 as shown in Table 1 is also sufficient because according to Schreiber et al. [27], researchers agreed on value of 10 respondents per estimated parameter rule is adequate. The number of parameters we used are 9 so 211 respondents is more than its needed. Gender, age distribution, frequency of attendance, kind of applications, type of internet network, internet cost, location, event intention duration, and event memorize are the parameters considered in this survey. For most studies, sample sizes of greater than 30 but less than 500 are suitable enough [28]. The survey is distributed only to closed WhatsApp groups consisting of academics, higher degree students, lecturers, and researchers.

**Table 1:** Response rate.

Total Population	Minimum Respondent MOE: 5%	Questioner returned
448	207	211

Source: Own editing, 2021.

### Analysis

Firstly, quantitative descriptive analysis with an explanatory inductive approach in the analysis process on the first phase. The data obtained will be read and displayed quantitatively at the same time given insight to help explain the phenomena found. However, we also do coding to help find out statistically of the questionnaire data obtained. In this research, the questions instrument is the various equipment and needs for the implementation of a webinar event as asked in our questionnaire. For the purposes of testing this questionnaire, the respondents was on various locations and have the same characteristics, namely academics or higher degree students, and faculty members. Mean and median are used to measure the central tendency to report. The mean is used to get an overall idea or picture of the data set and the median will give a more appropriate idea of the data distribution. By knowing the existing tendencies in the population group, we can generalize the behavior of a response. The median has the advantage of being determinable even if we don’t know the values of the scores at the extremes of the distribution [29].

In addition, an inductive technique was employed to build on emergent themes and develop a framework based on the findings of the initial study [30]. An induction activity was used to develop a generalization based on the analysis of a set of particulars [31]. This makes inductive approach as a generalization method. Generalization is the ability to make conclusions from the sample to the larger study population, and less frequently to other populations or cultures, was primarily envisaged in nomothetic terms [32]. Using an inductive technique involves gaining knowledge, comprehending phenomena, and building a new theory or model as new knowledge based on the new understanding [33]. So, based on the number of quantitative data collected, we also build an understanding based on previous research and references.

Secondly, we ran a variable test to see how different components of a sample’s characteristics affected the duration of endurance following the webinar. Gender, age, frequency, cost, and location to the intensity of the duration of participating in the webinar will be estimated using logistical analysis as independent variables in this study. The link between binary or ordinal answer probability and explanatory variables has been investigated using logistic regression analysis [34]. Conceptually, duration (DUR) is the dependent variable, while gender (GEN), age (AGE), frequency (FREQ), cost (COST), and place (PLACE) are independent variables.

There hasn’t been any information about a "ideal" webinar length before now [18]. So, we divide the duration into three groups of criteria, namely: 1–2 hours, 3–4 hours, more than 4 hours. The other variables and their parameter conditions that we have grouped can be seen in Table 2 below.

**Table 2:** The primary data types collected.

Symbol	Variable	Criterions
**DUR**	Duration of attending the webinar	1 = 1–2 hours
		2 = 3–4 hours
		3 = more than 4 hours
**GEN**	Gender	1 = male
		2 = female
**AGE**	Age	1 = 17–26 years
		2 = 27–36 years
		3 = 37–46 years
		4 = more than 46 years
**FREQ**	Frequency of attending webinars every week	1 = 1 time
		2 = 2–3 times
		3 = 4–5 times
		4 = more than 5 times
		5 = uncertain/ tentative
**COST**	Cost considerations	1 = cost-sensitive
		2 = non cost-sensitive
**PLACE**	Place to access the internet	1 = office/campus/public area
		2 = house

Source: Authors, 2021.

Furthermore, the binary logistic model was used to find the determinant factors on the duration of attending the webinar.

$$DUR=\beta_{0}+\beta_{1}GEN+\beta_{2}AGE+\beta_{3}FREQ+\beta_{4}COST+\beta_{5}PLACE+e$$

where: *β*_0−5_ = intercept and *e* = error

The binary logistic model seeks to address the flaws of the linear probability model, such as the fact that the probability might be less than zero or greater than one, and the partial effects of the explanatory variable remain constant [35]. The model is based on the cumulative logistic probability function. Thus, the dependent variable in this model is the probability of an event with a certain value from the explanatory variable [36]. Furthermore, if the data does not have a normal distribution or mutual covariance, the binary logistic model can be used instead [37].

To determine the independent variables and their interactions with the dependent variable, the binary logistic model is utilized. This model will describe and forecast the probability of the independent variable and dependent variable being combined [38]. The model is used to constrain probabilities to the [0, 1] intervals. If there is a function L, there is a likelihood [39]:

$$DUR\;(1)=\frac{e^{-1}}{{\left(1+e^{-1}\right)}^{2}},-\infty <1<\infty$$ (1)

The likelihood has the function of cumulative distribution:

$$\Lambda (1)=P[L\leq 1]=\frac{1}{1+e^{-1}}$$ (2)

Based on Table 2, the logistic probability in this study that the observed value DUR takes the value 1 is as follows.


$$P=P[L\leq \beta_{0}+\beta_{1}GEN+\cdots \ldots +\beta_{5}PLACE]$$ (3)

$$=\Lambda (\beta_{0}+\beta_{1}GEN+\cdots \ldots +\beta_{5}PLACE)$$ (4)

$$=\frac{1}{1+e^{-(\beta_{0}+\beta_{1}GEN+\cdots \ldots +\beta_{5}PLACE)}}$$ (5)

Therefore, the probability if y = 1 can be written as

$$p=\frac{1}{1+e^{-(\beta_{0}+\beta_{1}GEN+\cdots \ldots +\beta_{5}PLACE)}}$$ (6)

$$=\frac{\exp\left(\beta_{0}+\beta_{1}GEN+\cdots \ldots +\beta_{5}PLACE\right)}{1+exp(\beta_{0}+\beta_{1}GEN+\cdots \ldots +\beta_{5}PLACE)}$$ (7)

The probability if y = 0 is

$$1-p=\frac{1}{1+exp(\beta_{0}+\beta_{1}GEN+\cdots \ldots +\beta_{5}\mathrm{PLACE})}$$ (8)

Next, the Wald test is carried out which in principle is similar to the t-test

$t_{stat}=\frac{\beta_{k}-c}{se(\beta_{k})}$and it will be compared with *t_table_*: *t*(*N−K*) where the logit parameter estimator, N is the sample size, and K is the number of parameters that will be estimated. Apart from t-Wald, p-value can be used to determine the importance of each variable; the variable in this study will be significant if the p-value is less than 0.10.

## Results and discussion

We made a questionnaire with 9 questions which we distributed through WA groups containing faculty members, researchers, and university students only. We conducted the survey from 30 May 2020 to 7 June 2020. All 211 sample are represented by unique WhatsApp messenger numbers in the form of cell phone numbers. The results of our analysis are grouped into two parts:

### Descriptive analysis

#### Respondents characteristics

From the 211 respondents known to consist of 68 men and 143 women as seen on Table 3 below. The respondents taken from Whatssapp groups which are membered academics, researchers, and faculty members from Indonesia.

**Table 3:** Gender distribution of respondents.

Code	Classification	Mean		Median	Std. Deviation
		1,7		2,0	0,5
		Frequency	Percent	Valid Percent	Cumulative Percent
**1**	**Male**	68	32,2	32,2	32,2
**2**	**Female**	143	67,8	67,8	100,0
**Total**		211	100,0	100,0	

Source: Survey, 2020.

The age distribution of respondents relatively evenly started from the age of 17 years to over 46 years old. These conditions can be seen more clearly in Table 4 below.

**Table 4:** Age distribution of respondents.

Code	Range	Mean		Median	Std. Deviation
		2,2		2,0	1,0
		Frequency	Percent	Valid Percent	Cumulative Percent
**1**	**17–26 years**	56	26,5	26,5	26,5
**2**	**27–36 years**	79	37,4	37,4	64,0
**3**	**37–46 years**	46	21,8	21,8	85,8
**4**	**46+ years**	30	14,2	14,2	100,0
**Total**		211	100,0	100,0	

Source: Survey, 2020.

From the data above, the age distribution of respondents is even in almost all age ranges and describes the age of higher education graduates and faculty members in Indonesia. Mean (2,2) are between 27–36 years and some of 37–46 years.

#### Frequency of webinars attending

From the responses obtained it turns out that the majority of respondents or 38.9% do not necessarily attend webinars in a week, 34.6% of respondents attend 2 to 3 webinar events, 11.8% or 25 respondents take one webinar in a week, and only 6.2% follow more from 5 webinars in a week. This can be seen in Table 5 below.

**Table 5:** Frequency of webinars attending.

Code	Range	Mean		Median	Std. Deviation
		3,3		3,0	1,5
		Frequency	Percent	Valid Percent	Cumulative Percent
**1**	**1 time**	25	11,8	11,8	11,8
**2**	**2–3 times**	73	34,6	34,6	46,4
**3**	**4–5 times**	18	8,5	8,5	55,0
**4**	**5+ times**	13	6	6	61
**5**	**uncertain/ tentative**	82	39	39	100
**Total**		211	100,0	100,0	

Source: Survey, 2020.

Many respondents who only attended the webinar on an occasional/tentative basis described a serious issue in the involvement of knowledge dissemination. Some of the things that cause the low frequency are access to the digital system, computer literacy, limitations of technology, and the dependency in traditional systems [40]. However, this is a positive thing if it is considered that most respondents always try to participate in webinar events at least once a week. Ease of access to participate in webinars is a real thing during this pandemic, regardless of the technical issues that occur [41].

#### Application in following webinars

Zoom app is still the most dominant application used by webinar participants. We have not examined the use of the dominant Zoom application whether there is any relevance to the event organizer’s choice to use it. In other words, we don’t examine whether an event gets an audience because it uses the Zoom platform or other platforms. The second ranking for using the webinar application is the YouTube channel and is followed by the Google Meet application. Other applications are widely used by respondents but the numbers are not dominant. More detailed explanation can be seen in Table 6 below.

**Table 6:** Applications preferences.

Code	Application	Mean		Median	Std. Deviation
		2,0		2,0	0,5
		Frequency	Percent	Valid Percent	Cumulative Percent
**1**	**YouTube**	12	5,7	5,7	5,7
**2**	**Zoom**	183	86,7	86,7	92,4
**3**	**Google Meet**	10	4,7	4,7	97,2
**4**	**Others**	6	3	3	100
**Total**		211	100,0	100,0	

Source: Survey, 2020.

The others (2.8%) application used are Cisco WebEx, WhatsApp messenger, and Facebook. Zoom users who dominate are possible because it is the video conferencing application that was first popularized at the beginning of the pandemic in Indonesia. First impressions in marketing will tend to influence the choices made by consumers [42]. For small number of campuses, they may be used to using various video conferencing applications other than Zoom before the pandemic, but this does not apply to most scholars who previously used face to face meetings. Zoom also provides an opportunity for novice users without registration freely so that people in Indonesia like it [43]. Getting a product for free even with certain limitations is also preferred by consumers [44]. Controversial news that occurred on Zoom in 2000 about security issues also caught people’s attention [45]. In fact, controversy is an important part of a marketing process [46,47].

#### Network type and internet costs

We also find the fact that the use of cable/ fixed-based internet with stable data rates and fixed monthly payments are used by 46.9% of respondents. While the majority of respondents use a prepaid GSM card network with a data package purchase system. Only a small proportion of respondents use postpaid GSM cards (See Table 7).

**Table 7:** Network type.

Code	Type	Mean		Median	Std. Deviation
		1,6		2,0	0,6
		Frequency	Percent	Valid Percent	Cumulative Percent
**1**	**Fixed cable internet**	99	46,9	46,9	46,9
**2**	**Prepaid GSM**	101	47,9	47,9	94,8
**3**	**Post-paid GSM**	11	5,2	5,2	100,0
**Total**		211	100,0	100,0	

Source: Survey, 2020.

This finding is in line with the fact that the majority of respondents or 53.1% consider that the cost of internet data access is not a problem for them. In contrast, 46.9% considered internet costs to be a concern of respondents when they were about to take part in a webinar. We suspect that most likely respondents who feel concerned about these costs are prepaid GSM card users with a data package purchase system. Preference for concern for internet costs can be seen in Table 8 below.

**Table 8:** Internet costs concern.

Code	Sensitive	Mean		Median	Std. Deviation
		1,5		2,0	0,5
		Frequency	Percent	Valid Percent	Cumulative Percent
**1**	**Cost-sensitive**	99	46,9	46,9	46,9
**2**	**Non-cost sensitive**	112	53,1	53,1	100,0
**Total**		211	100,0	100,0	

Source: Survey, 2020.

The potential cost savings were one of the first motivating elements for management to push for the adoption and development of e-learning projects [48]. It should be noted that 46.9% of respondents feel that cost-sensitive is an obstacle for webinar organizers because they tend to give attention to the data package they have. Cost saving, in this case represented by network type and data package, found to positively influence purchase intention [49]. Indirect factors, in this case are the price and type of internet that is not provided by the organizer of this webinar, affecting purchase intention [50]. The webinars event or information dissemination through webinars need to be adjusted based on survey facts. Since the webinars in Indonesia are mostly free of charge so the organizers need to consider the price on the internet connection price that the audience must bear.

#### Webinars’s location accessing

Regarding the Work From Home/WFH policy in Indonesia, it is enforced to work or study from home for most of the scholar and faculty members. However, there are still those who access webinars from the office and campus because in Indonesia there are always staff or faculty members who enter the office occasionally. The data are obtained the conditions apply to respondents. It can be seen 81.5% of respondents stated that they followed a webinar from home. The remaining 15.6% are still following the webinar from their office or campus. Various other locations such as internet cafes, guard posts, vehicles, or public areas were chosen by webinar participants but the number was not significant. See Table 9.

**Table 9:** Location accessing webinar.

Code	Location	Mean		Median	Std. Deviation
		1,8		2,0	0,4
		Frequency	Percent	Valid Percent	Cumulative Percent
**1**	**Office/campus/public area**	36	17,1	17,1	17,1
**2**	**Home**	175	82,9	82,9	100,0
**Total**		211	100,0	100,0	

Source: Survey, 2020.

In order for a company to be successful, it must have a good distribution management system in place to get finished goods from the manufacturer to the end users [51]. Obviously, there will be differences in the treatment of potential customers, who are webinar attendees, when they attend events from home or from the office regarding the marketing strategy that will be carried out. Attending the webinar at home and in the office should be considered as form of distribution channel by the webinar event organizer. Thus, its distribution channels depend on the size of the sales network, or the number of sales facilities [52]. The place in the marketing concept for a webinar event can be assumed from what kind of location the event is accessed. It’s also linked to a good distribution procedure, which enables the company to accomplish the availability aim of delivering the product at the correct time and location [53].

#### Focused and webinar promotion

We try to find out the respondent’s loyalty in following the webinar event. Loyalty is determined in being able to follow in focus and able to follow events with optimal concentration subjectively in one event. Surprisingly we got the fact that most respondents or 85.3% were only able to stand and concentrate optimally within 1–2 hours in each webinar. Only 12.3% were able to participate optimally for 3–4 hours, 1.9% were able to attend 5–6 hours, and only 0.5% or 1 respondent was able to attend the webinar for more than 6 hours in each webinar session. The picture can be seen in Table 10 below.

**Table 10:** Duration in following the webinar event.

Code	In focus	Mean		Median	Std. Deviation
		1,2		1,0	0,4
		Frequency	Percent	Valid Percent	Cumulative Percent
**1**	**1–2 hours**	180	85,3	85,3	85,3
**2**	**3–4 hours**	26	12,3	12,3	97,6
**3**	**More than 4 hours**	5	2,4	2,4	100,0
**Total**		211	100,0	100,0	

Source: Survey, 2020.

This means that the audience only tends to focus for a short period of time so that insignificant content needs to be reduced, for example an opening speech(s) that is too long. Another challenge for event organizers is to develop the webinar presentation to increase the attention that drops after the first one to two hours in the event. Yet, face-to-face learning is more preferable compared to remote learning [54].

Another interesting habit fact about webinar promotion is the majority of participants or 44.5% of respondents are only able to remember 2 to 3 events in a week, 24.2% of respondents even only remember one event in a week, and 19.9% of respondents must be reminded before the webinar event will begin. More details can be seen in Table 11 below.

**Table 11:** Number of remembered webinars event.

Code	Event	Mean		Median	Std. Deviation
		2,5		2,0	1,4
		Frequency	Percent	Valid Percent	Cumulative Percent
**1**	**1 webinar**	51	24,2	24,2	24,2
**2**	**2–3 webinars**	94	44,5	44,5	68,7
**3**	**4–5 webinars**	21	10,0	10,0	78,7
**4**	**5+ webinars**	3	1	1	80
**5**	**Forgot & missed**	42	20	20	100
**Total**		211	100,0	100,0	

Source: Survey, 2020.

The more a consumer is exposed to advertising, the higher the probability of purchase because consumers are more likely to learn and retain advertisement content if the same message is repeated several times [55]. The same thing happens with webinar offers by the organizers because participants tend to forget about the schedule of events they want to join. The promotion of the webinar event also proved to be very strictly maintained. The audience’s memory which forgetting the event being offered so quickly that it had to be reminded a few moments before the event started become tricky issue for marketer. People, process and physical evidence that will be associated with webinar content getting mixed responses from the audience. Management can also take advantage of opportunities because almost 100 percent of scholars and faculty members attend webinars every week so that imaging webinar content as a useful issue is important since its usefulness proved to be a significant effect on consumer purchase intention [56]. Thus, this will determine the whole strategy for organizing committee in the battle for the audience’s attention.

The data on intention and habit above are important for webinar organizers in Indonesia to manage the duration of the webinar as intentions and habits become an important aspect in studying consumer behavior [57] which will then be discussed in the logistic regression analysis model.

### Logistics regression model

In this study, we examined the impact of five independent variables on the time spent watching the webinar. The study’s objectives are met using logit analysis. Table 12 shows the findings of the study.

**Table 12:** Examined independent variables.

Variable	Coef.	Std. Error	z-stat	Prob
**C**	1.193***	0.188	6.335	0.000
**Gender**	-0.112	0.064	-1.763	0.079
**Age**	0.071*	0.033	2.178	0.031
**Freq**	-0.028	0.020	-1.402	0.162
**Cost**	0.049	0.063	0.781	0.436
**Place**	0.012	0.061	0.203	0.839
**Log. likelihood**	-115.418		Schwarz crit.	1.246
**Prob. (LR stat)**	0.007		Hannan-Quinn crit.	1.189
			McFadden R-squared	0.074

Signif. codes: 0 ‘***’ 0.001 ‘**’ 0.01 ‘*’ 0.05 ‘.’ 0.1 ‘‘ 1.

Source: Author’s computation (2022).

Theoretically, the estimation of the binary logistic model did not require the use of multicollinearity, heteroscedasticity, and normality tests [58]. From the five independent variables used in this study, gender and age have a significant effect on the duration of attending the webinar. Meanwhile, the other 3 variables, frequency of attending webinars every week, cost considerations, and place to access the internet had no significant effect.

The gender variable has a significant effect on the dependent variable at a 10% level of significance. In comparison to other lengths, female respondents prefer to attend webinars that last 1 to 2 hours. On the other hand, male respondents prefer to attend webinars that go longer than two hours.

**Conclusion: Accept Hypothesis 1 (H1),** or gender has a significant effect on the duration of attending the webinar.

The age variable has a significant influence on the dependent variable at a 5% level of significance. Older respondents prefer to attend webinars with a duration of more than 4 hours. Meanwhile, younger respondents favor short-duration webinars (1 to 2 hours).

**Conclusion: Accept Hypothesis 2 (H2),** or age has a significant effect on the duration of attending the webinar.

Frequency of attending webinars every week did not have an affect the dependent variable. It has been shown to be statistically insignificant (p > 0.10). This indicates that frequency of attending webinars every week does not increase or decrease the likelihood of the duration of someone attending the webinar.

**Conclusion: Reject Hypothesis 3 (H3),** or frequency of attending webinars did not have a significant effect on the duration of someone attending the webinar.

Cost considerations did not have an affect the dependent variable. It has been shown to be statistically insignificant (p > 0.10). This indicates that cost considerations do not increase or decrease the likelihood of the duration of someone attending the webinar.

**Conclusion: Reject Hypothesis 4 (H4),** or cost considerations did not have a significant effect on the duration of someone attending the webinar.

Place to access the internet did not have an affect the dependent variable. It has been shown to be statistically insignificant (p > 0.10). This indicates that place to access the internet does not increase or decrease the likelihood of the duration of someone attending the webinar.

**Conclusion: Reject Hypothesis 5 (H5),** or place to access the internet did not have a significant effect on the duration of someone attending the webinar.

## Conclusion

The competitive market in selling webinar events in the pandemic era indicate a complex situation. The marketing management of an organization should think at least some related things to an adjusted marketing mix. Instead of spending large amounts on mass broadcasting through WhatsApp messenger, firms should do “behavioural targeting” or “micro targeting” which pay attention for highly targeted activities for select groups of consumers [55]. The marketing mix strategy can review competition in the market, so the 7p marketing mix is an significant part of a service plan which essential for optimum service delivery tools [59]. Further studies are needed to determine the webinar product marketing mix strategy.

We also found that gender and age influenced loyalty/intention/duration in participating in webinars. In general, women tend to attend webinars in shorter durations. Age also influences the duration of a person participating in the webinar. Young people are more likely to prefer webinars of shorter duration than older people. Of course, this will affect the delivery of content for those who want to hold a webinar event.

Many academics and institutions hold webinars to replace physical meetings as a phenomenon during the pandemic. Like a product, the webinar event turned out to be through very tight competition between organizers. In addition to intense competition, the webinar organizers also still have to create an atmosphere of events that can make participants attend and last a long time in participating in the event. People were influenced in different ways by the physical separation that was a part of the changes. Because some people’s behavior changes during the learning process, their personalities played a part [60]. Webinar event marketing strategies need to consider the facts of the field experienced by the audience and prospective audience. The facts obtained can be a consideration for parties who hold webinars and stakeholders in the dissemination of knowledge and information by online system. In general, we also conclude that knowledge dissemination using webinars still has severe limitations and challenges instead of physically replacing offline meetings.

## Limitations

This research is limited to focus on the academic community of higher education for their response to the promoted random webinars. We did not examine respondents with the lower level of education and non-academic audience. It will also be interesting if further research looks for reasons why women and young people tend not to stay long in webinars. Also, considering that older people tend to spend more time in front of screens during webinars, it’s critical to figure out why by using different research method (i.e.: mix-method). In addition, on other occasions it is important to know non-academic respondents or the public to know about the same conditions. Thus, the appropriate marketing strategy and content policies can be made to embrace audiences especially in information dissemination and another distance learning situations during the pandemic.
