# Correction: Webinar among Indonesian academics during Covid-19, embracing the audiences

**DOI:** 10.1371/journal.pone.0270440

**Published:** 2022-06-21

**Authors:** The PLOS ONE Staff

The second author’s name is spelled incorrectly. [1] The correct name is: Md Billal Hossain. The correct citation is: Priatmoko S, Hossain MB, Rahmawati W, Winarno SB, Dávid LD (2022) Webinar among Indonesian academics during Covid-19, embracing the audiences. PLoS ONE 17(3): e0265257. https://doi.org/10.1371/journal.pone.0265257. The publisher apologizes for the error.
